# Strategies and resources used by public health units to encourage COVID-19 vaccination among priority groups: a behavioural science-informed review of three urban centres in Canada

**DOI:** 10.1186/s12889-025-21342-1

**Published:** 2025-01-31

**Authors:** Tori Langmuir, Mackenzie Wilson, Nicola McCleary, Andrea M. Patey, Karim Mekki, Hanan Ghazal, Elizabeth Estey Noad, Judy Buchan, Vinita Dubey, Jana Galley, Emily Gibson, Guillaume Fontaine, Maureen Smith, Amjad Alghamyan, Kimberly Thompson, Jacob Crawshaw, Jeremy M. Grimshaw, Trevor Arnason, Jamie Brehaut, Susan Michie, Melissa Brouwers, Justin Presseau

**Affiliations:** 1 Centre for Implementation Research, Methodological and Implementation Research Program, Ottawa Hospital Research Institute, Ottawa, ON Canada; 2 School of Epidemiology and Public Health, University of Ottawa, Ottawa, ON Canada; 3 School of Rehabilitation Therapy, Queen’s University, Kingston, ON Canada; 4 Ottawa Public Health, Ottawa, ON Canada; 5 Peel Public Health, Mississauga, ON Canada; 6 Toronto Public Health, Toronto, ON Canada; 7 Department of Medicine, University of Ottawa, Ottawa, ON Canada; 8 Citizen Engagement Co-Lead, Ottawa, ON Canada; 9 University of Ottawa, Ottawa, ON Canada; 10 The Hospital for Sick Children (SickKids), Toronto, ON Canada; 11 Centre for Behaviour Change, University College London, London, UK; 12 School of Psychology, University of Ottawa, Ottawa, ON Canada; 13 Department of Psychology, Concordia University, Montréal, QC Canada; 14 Child Health Evaluative Sciences Program, The Hospital for Sick Children - Research Institute, Toronto, ON Canada; 15 Institute of Health Policy, Management and Evaluation, University of Toronto, Toronto, ON Canada; 16 Department of Medicine, Quality and Safety, IWK Health, Halifax, NS Canada; 17 Kirby Institute, UNSW Sydney, Kensington, NSW Australia; 18 Ingram School of Nursing, Faculty of Medicine and Health Sciences, McGill University, Montréal, QC Canada; 19 Centre for Clinical Epidemiology, Lady Davis Institute for Medical Research, Sir Mortimer B. Davis Jewish General Hospital, CIUSSS West-Central Montréal, Montréal, QC Canada; 20 Centre for Nursing Research, Sir Mortimer B. Davis Jewish General Hospital, CIUSSS West-Central Montréal, Montréal, QC Canada

**Keywords:** COVID-19 vaccine, Booster dose, Behavioural science, Health psychology, Priority groups, Equity deserving, Community engagement, Vaccine uptake

## Abstract

**Background:**

Ensuring widespread COVID-19 vaccine uptake is a public health priority in Canada and globally, particularly within communities that exhibit lower uptake rates and are at a higher risk of infection. Public health units (PHUs) have leveraged many resources to promote the uptake of recommended COVID-19 vaccine doses. Understanding barriers and facilitators to vaccine uptake, and which strategies/resources have been used to address them to date, may help identify areas where further support could be provided. We sought to identify the strategies/resources used by PHUs to promote the uptake of the first and third doses of the COVID-19 vaccine among priority groups in their jurisdictions. We examined the alignment of these existing strategies/resources with behavioral science principles, to inform potential complementary strategies/resources.

**Methods:**

We reviewed the online and in-person strategies/resources used by three PHUs in Ontario, Canada to promote COVID-19 vaccine uptake among priority groups (Black and Eastern European populations, and/or neighbourhoods with low vaccine uptake or socioeconomic status). Strategies/resources were identified from PHU websites, social media, and PHU liaison. We used the Behaviour Change Techniques (BCT) Taxonomy – which describes 93 different ways of supporting behaviour change – to categorise the types of strategies/resources used, and the Theoretical Domains Framework – which synthesises 14 factors that can be barriers or facilitators to decisions and actions – to categorise the barriers and facilitators addressed by strategies/resources.

**Results:**

PHUs operationalised 21 out of 93 BCTs, ranging from 15 to 20 BCTs per PHU. The most frequently operationalised BCTs were found in strategies/resources that provided information about COVID-19 infection and vaccines, increased access to COVID-19 vaccination, and integrated social supports such as community ambassadors and engagement sessions with healthcare professionals. Identified BCTs aligned most frequently with addressing barriers and facilitators related to Knowledge, Environmental context and resources, and Beliefs about consequences domains.

**Conclusion:**

PHUs have used several BCTs to address different barriers and facilitators to COVID-19 vaccine uptake for priority groups. Opportunities should be pursued to broaden the scope of BCTs used (e.g., operationalizing the *pros and cons* BCT) and barriers/facilitators addressed in strategies/resources for ongoing and future COVID-19 vaccine uptake efforts among general and prioritised populations.

**Supplementary Information:**

The online version contains supplementary material available at 10.1186/s12889-025-21342-1.

## Background

Since the COVID-19 vaccine rollout began in Canada in December 2020, the strong initial uptake of the first and second doses (primary series) tapered off, and subsequent doses have not seen the same rates of uptake as the primary series. In Canada, 90.5% of Canadians (ages 18+) have received at least one dose, 88.9% have completed their primary series of two doses, and 51.5% have completed the primary series plus one dose (as of June 2023; [1]). Vaccination, including doses after the primary series (i.e., “booster doses”), helps to reduce the impact of COVID-19 [2, 3], which has caused 6.9 million deaths globally (as of June 2023; [4]). With persisting risks of new variants of concern emerging, the potential of additional COVID-19 waves, and waning effectiveness of vaccines over time, on-going vaccination concordant with evidence-based guidelines remains a fundamental tool in preventing morbidity and mortality associated with COVID-19 infection [5, 6]. Throughout the COVID-19 pandemic and in general, municipal public health units (PHUs) in Ontario, Canada (and many other jurisdictions worldwide) have been centrally positioned in supporting the citizens in their jurisdictions to decide to and get vaccinated through local campaigns, facilities, and programs. This is consistent with the role that PHUs have more broadly in the prevention and management of infectious diseases in the community.

Behavioural science has an important role in public health crises such as a pandemic by providing tools to help understand and support taking up new and maintaining existing health-protective behaviours like following public health and social measures to reduce the spread of COVID-19, such as masking, physical distancing, and vaccination [7]. As the COVID-19 virus continues to mutate and remains a public health concern, a particularly pertinent application of the behavioural sciences is to identify factors that may influence an individual’s decision or action to get vaccinated or not and encourage uptake of eligible doses of COVID-19 vaccine [8].

There are several evidenced tools and frameworks available from the behavioural sciences that can provide the foundation for identifying barriers and facilitators to engaging in health-protective behaviours, including vaccination. The Theoretical Domains Framework (TDF; [9, 10]) synthesises 33 theories of behaviour and behaviour change into 14 domains (Table 1) and has been used widely to explore barriers and facilitators to the implementation of evidence-based practices in various health disciplines [9]. Originally developed to synthesise theories of behaviour and behaviour change to understand health professional behaviour, the TDF has been applied to assess health behaviours and interventions with patients and the public [11–13]. This extension of the TDF is consistent with much of the theory and models that inform the TDF, many of which have largely been developed originally to understand the behaviour of patients and the public. The TDF is a more fine-grained categorisation construct that aligns with the three key overarching capability, opportunity, and motivation factors within the Behaviour Change Wheel [14].

**Table 1:** Theoretical domains from the theoretical domains Framework (TDF) and their descriptions

Theoretical domain	Definitions^1^
Knowledge	Existing procedural knowledge, knowledge about guidelines, knowledge about evidence and how that influences what individuals do
Skills	Competence and ability about the procedural techniques required to perform the behaviour
Social/professional role and identity	Boundaries between professional groups (i.e., is the behaviour something the individual is supposed to do or someone else’s role? )
Beliefs about capabilities	Perceptions about competence and confidence in doing the behaviour and how that influences their behaviour
Optimism	Whether the individual’s optimism or pessimism about the behaviour influences what they do
Beliefs about consequences	Perceptions about outcomes, advantages and disadvantages of performing the behaviour and how that influences whether they perform the behaviour
Reinforcement	Previous experiences that have influenced whether or not the behaviour is performed
Intentions	A conscious decision to perform a behaviour or a resolve to act in a certain way
Goals	Priorities, importance, commitment to a certain course of actions or behaviours
Memory, attention, and decision processes	Attention control, decision-making, memory (i.e., is the target behaviour problematic because people simply forget? )
Environmental context and resources	How factors related to the setting in which the behaviour is performed (e.g., people, organisational, cultural, political, physical and financial factors) influence the behaviour
Social influences	External influence from people or groups to perform or not perform the behaviour How the views of colleagues, other professions, patients and families, and doing what you are told, influence the behaviour
Emotion	How feelings or affect (positive or negative) may influence the behaviour
Behavioural regulation	Ways of doing things that relate to pursuing and achieving desired goals, standards or targets Strategies the individuals have in place to help them perform the behaviour Strategies the individuals would like to have in place to help them

^1^ adapted from Grimshaw and colleagues [15]

The Behaviour Change Technique Taxonomy (BCTT; [16]) consists of 93 evidence-based Behaviour Change Techniques (BCTs) hierarchically clustered in 16 groups which can be mapped onto empirically linked TDF domains to identify which types of BCTs may be best suited to address specific barriers or facilitators identified within a TDF domain [16]. For example, BCTs with social components may be mapped onto the Social Influences domain, and BCTs with environmental components may be mapped onto the Environmental Context & Resources domain. It is common for multiple BCTs to be mapped onto TDF domains and vice-versa. The Theory and Techniques Tool facilitates the mapping of linked domains and BCTs, and links evidence from other behavioural science frameworks, via an online matrix [17]. It includes a “heat map” visual style to indicate the strength of each link, with citations for supporting literature [17]. Together, the TDF and the BCTT can be used to develop interventions to address the barriers and facilitators to a given behaviour using evidence-based techniques which represent the ‘active ingredients’ (i.e., salient and modifiable factors) in behaviour change [16, 18].

Vaccine acceptance and uptake data from the first two years of the COVID-19 pandemic show that members of some equity-deserving groups, including those experiencing racial, ethnic, and socioeconomic disparities, had lower rates of acceptance and uptake. For example, there is evidence to suggest that individuals from Black, Hispanic, and Indigenous communities were less likely to express vaccine acceptance than white individuals [19–22], but vaccine hesitancy has also been found among white populations of various demographics, including Eastern European, such as Ukrainian refugees [23–25]. In each group, commonly reported barriers to COVID-19 vaccination include mistrust and misinformation [20, 22–25]. This suggests that some equity-deserving groups may experience unique barriers to getting vaccinated that approaches taken to date by PHUs have not sufficiently addressed [26, 27]. A 2021 behavioural science living evidence synthesis by Crawshaw and colleagues [21] using the TDF to categorise barriers and facilitators to COVID-19 vaccination found that of the 175 studies that reported on factors influencing acceptance and uptake to that point, 34 focused on equity-deserving groups, including Black and low socioeconomic status populations. Within these, the authors identified three predominant TDF domains: Social influences, Beliefs about consequences and Environmental context and resources. Of the barriers identified, concerns about vaccine safety, efficacy, and necessity (Beliefs about consequences), mistrust in the government/public health response to COVID-19 (Social influences), and access issues in terms of time, convenience, and cost (Environmental context and resources) were especially common among Black populations. Facilitators included having access to and trust in reputable scientific/non-scientific information sources about COVID-19 and COVID-19 vaccines (Environmental context and resources) and feeling at high-risk for COVID-19 infection (Beliefs about consequences) [21]. As none of the literature reviewed by Crawshaw and colleagues [21] used a comprehensive framework such as the TDF to identify barriers among these groups, it is possible that some barriers were under-represented for some equity-deserving communities. Additional theoretical domains have been implicated in the literature as barriers or facilitators to vaccine acceptance and/or uptake in the general population, including Knowledge (and gaps in knowledge), Social/professional role and identity (seeing vaccination as a collective responsibility), Reinforcement (getting other vaccines in the past), and Emotion (emotional distress such as anxiety or depression) [21].

Further work to identify existing PHU strategies/resources using behavioural science frameworks may help to systematically describe (i) which strategies/resources have already been used and (ii) which barriers to vaccine uptake have therefore already been focused on. Mapping this onto what is known about barriers specifically faced by equity-deserving groups may enable identifying remaining gaps (in terms of barriers currently not being addressed) and highlighting opportunities for existing strategies/resources to be optimised or new strategies/resources to be deployed which are best suited for these groups. Using a common framework to classify and understand which strategies/resources have been used by PHUs to address specific barriers to vaccination in a given jurisdiction could help to establish a common description of strategies/resources across jurisdictions and to identify opportunities for optimising approaches to best serve the needs of specific priority groups.

We aimed to (a) identify how three PHUs in Ontario (Canada) promoted uptake of doses of the COVID-19 vaccine amongst groups with relatively lower uptake of COVID-19 vaccines (i.e., priority groups), and (b) use behavioural science tools to classify existing strategies/resources used by these PHUs and identify the barriers and facilitators to vaccination that these strategies are designed to address.

This research informed a broader research study (the OPTimise Platform; [28]) which partnered with local community members and PHUs to identify factors that influence COVID-19 vaccine acceptance and uptake for prioritised groups and make evidence-based recommendations for strategies/resources to support vaccination locally within these groups. The project involved three PHUs with which the research team had established pre-existing connections during the pandemic (in Ottawa, Peel Region, and Toronto) that each prioritised a specific group in their communities and dose of COVID-19 vaccination for which they would benefit from recommendations to address behavioural uptake. Local groups and a COVID-19 protective behaviour (e.g., vaccination, masking, or social distancing) were prioritised by PHUs according to guidelines provided to PHUs by the research team. These included lower uptake of behaviour compared with other jurisdictions, previous engagement by PHU with limited results on behaviour, underserved or equity-deserving groups, and aligns with PHU concerns trending over the coming months. For more information on the prioritisation process, please see Fontaine and Smith et al. (2024).

## Method

### Prioritising groups and COVID-19 protective behaviours

In January 2022, each PHU provided the research team with (1) a priority group in their jurisdiction and (2) a COVID-19 protective behaviour. Each decided on a variation of COVID-19 vaccination (i.e., first or third dose). The Ottawa PHU prioritised uptake of the third dose of the COVID-19 vaccine among individuals ages 18 + living in five low socioeconomic status neighbourhoods. The Peel PHU prioritised uptake of the first dose of the COVID-19 vaccine among individuals between the ages of 30 and 49 in Eastern European communities (specifically Polish, Ukrainian, and Russian). The Toronto PHU prioritised uptake of the first dose among individuals ages 18 + from five neighbourhoods with the lowest rates of vaccination who are members of Black communities.

### Design

We formally assessed online, publicly available information, resources, messages and in-person activities (defined as “strategies/resources” for the purpose of this study) used by the PHUs to promote COVID-19 vaccination (first or third doses). Our findings were coded according to BCTs [16] and mapped to the TDF [17, 18].

### Procedure

We conducted an online search between March 11, 2022, and May 11, 2022, and included PHU strategies/resources which were available from January 1, 2021 – May 11, 2022 (i.e., beginning from when the PHUs began COVID-19 vaccine strategy/resource rollout). The online search used Google, websites of the three PHUs, and their social media pages across four predominant platforms at the time (Facebook, Twitter, YouTube, and Instagram). Search terms included behaviour-related keywords such as “COVID-19”, “vaccine”, and “booster”, and population-related terms such as “Black”, “Ukrainian”, and “Newcomer”. A full list of search terms can be found in Appendix 1. Strategy/resource findings were checked for completeness and accuracy by co-authors working at each PHU (KM, HG, VD, LEN) in Winter 2023. We also asked PHUs to identify any strategies/resources that may not have been published online, and whether/how they were tailored to priority groups.

### Eligibility criteria

#### Data sources

Data included abstracted findings about strategies/resources from publicly available PHU webpages and social media, including text, photos, videos, infographics, downloadable materials, interactive maps, tools, calendars, and any other online media which pertained to the prioritised dose. Second-order sources, defined as strategies/resources linked or embedded within findings that did not originate from the PHU, such as provincial or federal webpages, were included. Data on unpublished strategies/resources were obtained from PHUs through liaison with staff at each PHU who were involved in leading COVID-19 vaccine initiatives, including those who served the identified priority groups.

#### Data abstraction

Data were abstracted from strategy/resource findings by two reviewers (TL and JG). Only data tailored to the priority behaviour (i.e., first or third dose) were abstracted. Strategies/resources promoting COVID-19 vaccination in general as opposed to the first dose specifically were assumed to implicitly include promotion of the first dose. Using Microsoft Excel, we abstracted data from webpages and social media including source, platform, timestamp, deviation, mode of delivery, description, tailoring to priority behavior and group (implicit/explicit geographic or cultural tailoring such as language, visuals, or localization), language(s), and rationale. A complete list of data abstraction variables and definitions can be found in Appendix 2.

### Data analysis

To guide coding and inform the larger OPTimise Platform study, a project subgroup focused on project methods (AMP, NM, JP, MW, TL) identified potentially relevant operationalisations of the 93 BCTs in support of COVID-19 first or third dose vaccination, with added considerations for equity-deserving groups. Strategy/resource findings from each PHU were independently coded by the first reviewer (TL) to BCTs and mapped to empirically linked TDF domains using the Theory and Techniques Tool [17]. To ensure results were based on empirical evidence, domains were only included in the domain frequency analysis if they were conclusively linked (i.e., not inconclusively, non-links, or no evidence) to an identified BCT. However, to maximise descriptiveness of the BCTs that have been implemented in real-world settings, all identified BCTs were included in BCT frequency analyses, regardless of links to the TDF. Findings from all three PHUs were merged into a master document to examine overall trends. Coding was validated by the second reviewer (MW) and disagreements were resolved in consultation with an expert (NM).

## Results

### BCTs

Twenty-one unique BCTs were operationalised across the identified strategies/resources used by the three PHUs. Table 2 shows the operationalised BCTs for identified PHU strategies and resources (behaviour-specific and population-specific), and their frequencies.

**Table 2:** Behaviour Change techniques (BCTs) and frequencies in public health unit (PHU) strategies/resources for COVID-19 vaccination

BCTs	PHU Strategies and resources	Frequency across PHUs
3.1. Social support (unspecified)	Videos of community ambassadors, HCPs, and general public sharing personal experiences and reasons why they got vaccinated, clinics and information sharing at community hubs and events (e.g., faith centres, hair salons), encouraging discussions with peers and community members about getting vaccinated, engagement sessions with health experts of different cultural backgrounds, neighbourhood vaccine engagement and outreach teams	40
3.2. Social support (practical)	Clinic transportation services and vouchers, low-barrier clinics with extended hours/childcare/accessibility supports (e.g., ramps, no insurance needed), clinics that can be requested and led by community members, neighbourhood vaccine engagement and outreach teams, town halls and webinars with HCPs, multilingual community ambassadors	45
3.3. Social support (emotional)*	Clinic companions for mitigating needle fear	1
4.1. Instruction on how to perform behaviour	Appointment booking links, clinic locations and access information (e.g., walk-in, parking, transit routes), how/where to get vaccinated webpages (e.g., search tool to find nearby clinic), eligibility information	62
5.1. Information about health consequences	Information about benefits of vaccination and risks of COVID-19 infection	76
5.2. Salience of consequences	Videos of community members describing impact of COVID-19 infection on themselves/loved ones (as reasons for getting vaccinated)	3
5.3. Information about social and environmental consequences	“Protect others/loved ones” messaging, describing impact on community and disproportionate impact on Black communities	8
6.1. Demonstration of the behaviour	“I received my first dose” graphic for sharing on social media; video testimonials of people at clinics during/after vaccination	7
6.2. Social comparison	Sharing vaccination coverage rates, HCPs sharing why they got vaccinated, videos of community members talking about why they got or changed their mind about getting the vaccine, “I received my first dose” graphic for sharing on social media	16
6.3. Information about others’ approval	Videos, town halls, and engagement sessions with HCPs providing information and answering questions, community ambassadors, members, and HCPs sharing why they got vaccinated and encouraging others to get vaccinated, “I received my first dose” graphic for sharing on social media	27
7.1. Prompts/cues	Social media reminder posts, banners at the top of webpages with reminders about vaccination, physical and digital ad campaigns, auto-calls and mass text campaigns, flyers, community ambassador door-knocking programs	29
8.6. Generalisation of target behaviour*	Combining with influenza vaccine reminders, messaging, and clinics	1
9.1. Credible source*	Videos, town halls, and engagement sessions with HCPs answering questions and discussing safety and benefits of vaccination, community leaders and HCPs talking about why they got vaccinated	27
10.1. Material incentive (behaviour)	Incentive programs (financial voucher) in shelter settings and for precariously housed populations	1
10.2. Material reward (behaviour)	Financial voucher provided to those who get the vaccine in shelter settings and for precariously housed populations	3
10.6. Nonspecific incentive	“Getting back to the things we love/normal” messaging	3
11.2. Reduce negative emotions	Conversations, panels, and information sessions with community ambassadors and HCPs to address concerns about vaccination	12
11.3. Conserve mental resources	Multilingual promotional materials (e.g., shared in person or via WhatsApp) and community ambassadors	6
12.1. Restructuring the physical environment	Pop-up and community clinics (e.g., clinics at shopping centres, transit stations), mobile clinics (e.g., busses, vans), clinics with extended hours, childcare, walk-ins, or other access supports	41
12.2. Restructuring the social environment	Community clinics, mobile clinics, pop-up clinics that can be requested and led by community members, social media ad campaigns and engagement sessions (e.g., WhatsApp), clinics and information sharing at community hubs and events (e.g., faith centres, hair salons)	20
12.5. Adding objects to environment	Pop-up and community clinics (e.g., shopping centres, transit stations), mobile clinics (e.g., busses, vans)	38

Note. HCPs = health care professionals

*Not conclusively linked to a TDF domain at time of analysis

Overall, the most frequently operationalised BCTs across all PHU strategies/resources were: *information about health consequences*, *instruction on how to perform behaviour*, *social support (unspecified)*,* social support (practical)*, *restructuring the physical environment*, and *adding objects to the environment*.

Use of *information about health consequences* refers to both negative and positive consequences (i.e., effects or results) of engaging in a behaviour and is a highly relevant BCT for individuals who are at the decision-making stage about whether they should receive a dose of the COVID-19 vaccine or not. It was the most frequently identified BCT across PHU strategies/resources. All PHUs integrated information sharing throughout their online and in-person strategies/resources, through various communication channels including links and text on websites, posts and videos on social media, and information sessions and outreach both in-person (e.g., door knocking, mailing flyers) and on social media (e.g., WhatsApp).

*Instruction on how to perform behaviour* was operationalised by all PHUs through resources that provided instructions or information on how to receive a dose of a COVID-19 vaccine. This included links, tools, and information for determining dose eligibility, locating, booking, and accessing COVID-19 vaccines, such as instructions on which public transit routes to take and when to access specific clinics in a local area. Many of these strategies/resources were found online and several instances were second-order links to provincial webpages with appointment booking tools.

Two BCTs for types of *social support*, *unspecified* and *practical*, were also frequently used across PHU strategies. *Social support (practical)* describes any form of practical help (e.g., from friends, relative, colleagues, or staff) in performing a behaviour. This was operationalised by PHUs through strategies that addressed barriers to accessing vaccination or getting information about vaccination, such as free transportation to/from clinics, in-home vaccination programs, neighbourhood and community outreach/engagement with multilingual staff/volunteers, and access supports at clinics (e.g., extended hours, free childcare, walk-ins available). *Social support (unspecified)* more broadly encompasses any form of social support to support performance of a behaviour, such as counselling and encouragement. This was operationalised by PHUs largely through their respective community outreach/engagement campaigns, involving community members and healthcare professionals sharing their experiences with COVID-19 vaccination, answering questions, providing information, and encouraging others to get vaccinated. For all PHUs, community-based outreach was (and continues to be) conducted both online (e.g., videos on YouTube and PHU websites, WhatsApp), and in-person (e.g., events at community hubs such as churches and libraries, door knocking).

Another two frequently identified BCTs were related to changes in or additions to the physical environment implemented by PHUs to increase or facilitate access to COVID-19 vaccines. *Restructuring the physical environment* and *adding objects to the environment* describe exactly that and were both operationalised by all PHUs through pop-up vaccine clinics in local hubs, such as malls, schools, workplaces, transit stations, parking lots, or community centres. The former included accessibility supports at clinics such as those mentioned previously and was therefore operationalised slightly more frequently than the latter. A related but less frequently operationalised BCT is *restructuring the social environment*, used by PHUs in resources/strategies which involved offering information and engagement sessions with healthcare professionals at social and community hubs both in-person (e.g., community centres) and online (e.g., Facebook, WhatsApp).

Some PHU strategies/resources operationalised several BCTs. For example, all PHUs used a combination of YouTube video campaigns, town halls, and webinars with healthcare professionals of various cultural backgrounds discussing vaccines and addressing concerns about safety and development in different languages. The delivery of strategies/resources multilingually operationalises the BCT *conserve mental resources* by communicating in a language that may be more accessible and/or easily understood. The presence of an expert or authority figure expressing approval of a behaviour (in this case, urging individuals to get vaccinated against COVID-19) operationalises the BCTs *information about others’ approval* and *credible source*. The social aspect of these engagement strategies/resources operationalise the BCTs *social support (unspecified) and social support (practical)*, and the informational aspect operationalises the BCT *information about health consequences*, as well as *information about social and environment consequences* when the impact of COVID-19 on specific communities is discussed. When these health professionals and ambassadors address concerns about vaccines, such as side-effects or development, they operationalise the BCT *reduce negative emotions*. Some of these campaigns also included community members and ambassadors sharing their experiences with and reasons for getting the COVID-19 vaccine, often including real stories of how COVID-19 infections impacted themselves and/or loved ones, which operationalises the BCTs *salience of consequences* and *social comparison*. Sometimes, these videos were filmed while individuals received a dose of the COVID-19 vaccine on camera, which operationalises the BCT *demonstration of behaviour*. The BCT *social comparison* was also operationalised a few times through social media posts and webpages sharing vaccine coverage/uptake data (e.g., “50% of our city has received their first dose”), which draws attention to others’ vaccination behaviour to allow comparison with one’s own vaccination behaviour.

The BCT *prompts/cues* describes anything that reminds the individual about performing the intended behaviour and was operationalised somewhat frequently across many different types of strategies/resources. Examples include an eye-catching yellow banner at the top of all Toronto PHU webpages, reminding residents to get vaccinated against COVID-19 with links for more information, social media posts from all PHUs reminding the public about COVID-19 vaccines, digital and physical advertising campaigns (e.g., on social media, busses, flyers in the mail), and vaccine engagement/outreach through community partners (e.g., door-knocking, community events).

BCTs which were least frequently operationalised by PHUs were *social support (emotional)*, *salience of consequences*, *generalisation of target behaviour*, and *material incentive (behaviour)*, *material reward (behaviour)*, and *nonspecific incentive*.

*Social support (emotional)* was operationalised once by a Toronto vaccine clinic that promoted the availability of nurses and support persons trained to mitigate needle fear. Generalisation of target behaviour was also operationalised once in Toronto by including the promotion of COVID-19 vaccines alongside the regular promotion of influenza vaccines. Two related BCTs, *material incentive* and *material reward*, were operationalised by financial incentive programs implemented in housing shelters and street outreach campaigns, where individuals were offered money or vouchers after receiving a dose of the COVID-19 vaccine. The incentive in this case is the money/vouchers that are offered to individuals if they compete the behaviour (receiving a dose of the COVID-19 vaccine), and the reward is the delivery of the money/voucher after completion of the behaviour. Lastly, a third BCT, *nonspecific incentive*, was operationalised through PHU strategies/resources that encouraged individuals to get vaccinated in order to “return to normal”, “get back to the things we love”, and other similar messaging that incentivizes COVID-19 vaccination without specifying a clear reward.

A few strategies/resources which were included in analysis did not operationalise any BCTs that directly supported vaccination behaviours, such as translation options on PHU webpages and funding and member information for vaccine engagement teams.

### Behaviour-specific

A total of 15–20 BCTs were operationalised within 39–79 behaviour-specific strategies/resources per PHU (Table 3). All strategies/resources included in analysis were behaviour-specific.

**Table 3:** Behaviour Change Techniques (BCTs) identified in strategies/resources used by public health units (PHUs)

Strategies/resources	Total number of strategies and resources	Total number of BCTs used	BCT Taxonomy codes (see Table 2)
**Behaviour-specific**			
Ottawa (3rd dose)	39	15	3.1, 3.2, 4.1, 5.1, 5.2, 5.3, 6.1, 6.2, 6.3, 7.1, 9.1, 11.3, 12.1, 12.2, 12.5
Peel Region (1st dose)	49	17	3.1, 3.2, 4.1, 5.1, 5.3, 6.1, 6.2, 6.3, 7.1, 9.1, 10.2, 10.6, 11.2, 11.3, 12.1, 12.2, 12.5
Toronto (1st dose)	79	20	3.1, 3.2, 3.3, 4.1, 5.1, 5.2, 5.3, 6.1, 6.2, 6.3, 7.1, 8.6, 9.1, 10.1, 10.2, 10.6, 11.2, 12.1, 12.2, 12.5
**Population-specific**			
Ottawa (low SES neighbourhoods)	13	10	3.1, 3.2, 4.1, 5.1, 6.2, 7.1, 11.3, 12.1, 12.2, 12.5
Peel (Eastern European communities)	21	12	3.1, 3.2, 4.1, 5.1, 5.3, 6.2, 7.1, 11.2, 11.3, 12.1, 12.2, 12.5
Toronto (Black communities in low uptake neighbourhoods)	31	17	3.1, 3.2, 4.1, 5.1, 5.2, 5.3, 6.1, 6.2, 6.3, 7.1, 9.1, 10.1, 10.2, 11.2, 12.1, 12.2, 12.5

Note. SES = socioeconomic status

Across PHUs, strategies/resources usually focused on promoting COVID-19 vaccination in general rather than by specific dose (i.e., “get vaccinated against COVID-19” instead of “get your first/third dose of the COVID-19 vaccine”). In a few strategies/resources, Toronto and Peel PHUs specifically mentioned a first dose, particularly after COVID-19 vaccines became available (e.g., “first dose is available”, “over 75% first dose rate”). We found a few social media posts from the Ottawa PHU promoting the availability of booster doses at specific clinics. The Ottawa PHU also had several in-person strategies/resources promoting booster doses specifically, such as mobile clinics offering booster doses at community locations (e.g., aging in place builds, malls, schools, long-term care homes) and community partnerships (e.g., with religious leaders, residents) for peer-to-peer information sharing about the third dose. The Toronto and Peel PHUs employed similar community-based in-person strategies targeting COVID-19 vaccine uptake.

### Population-specific

A total of 10–17 BCTs were operationalised within 13–31 population-specific strategies/resources per PHU. Overall, we found fewer strategies/resources from all PHUs that were tailored to the priority groups, and fewer BCTs. Compared to behaviour-specific BCTs, population-specific BCTs tended to be less frequently operationalised, but the BCTs and how they were operationalised tended to be similar.

In Ottawa, a few mentions of clinic locations/hours and vaccine availability within the prioritised group (neighbourhoods) were found in online strategies/resources, operationalising the BCT *instruction on how to perform behaviour*. We found more population-specific strategies/resources and BCTs which were delivered in-person than online, such as pop-up clinics and information sharing and outreach in the priority neighbourhoods. Of note, because the Ottawa PHU prioritised neighbourhood groups, we explored their tailoring at a geographic level only, whereas Peel and Toronto prioritised cultural/ethnic/age groups and/or neighbourhoods, thus potentially providing more opportunities for identifying tailored strategies/resources.

In Peel, a few population-specific strategies/resources were found on the PHU website. There was a sidebar option for translation, including into Russian, Polish, and Ukrainian (implicit tailoring), and a few documents in Russian and Polish languages with information about COVID-19 vaccines (e.g., questions and answers). No strategies/resources tailored to the Peel prioritised age group (30–49 years) were found. A few in-person strategies were tailored to Eastern European populations including outreach via community ambassadors to faith-based institutions (e.g., churches, cultural centres), businesses, and community events (e.g., cultural celebrations). However, the Peel PHU noted that these outreach attempts were sometimes turned down by Eastern European organisations and groups, who explained that they would prefer to remain neutral on the topic of COVID-19 vaccination.

In Toronto, the PHU’s tailored strategies/resources were primarily YouTube videos featuring healthcare professionals and scientific experts from Black communities (*credible source*) discussing information about COVID-19 vaccines (*information about health consequences*) and the impact of COVID-19 on Black communities (*information about social and environment consequences*), addressing concerns about vaccine development, side-effects, and adverse reactions (*reduce negative emotions*), and encouraging all individuals including members of Black communities to get vaccinated against COVID-19 (*unspecified social support*). These videos often featured speakers from diverse cultural backgrounds, in different languages (*practical social support* and *conserve mental resources*). The Toronto PHU developed focused, in-person initiatives to support vaccination among Black communities during COVID-19, such as the Black Scientists’ COVID-19 Task Force and the Black Vaccine Engagement Team which operationalise the same BCTs. Some strategies/resources tailored to the prioritised neighbourhoods were found, but we found fewer BCTs or strategies/resources tailored to members of Black communities who live in the prioritised neighbourhoods.

### TDF domains

**Fig. 1:**
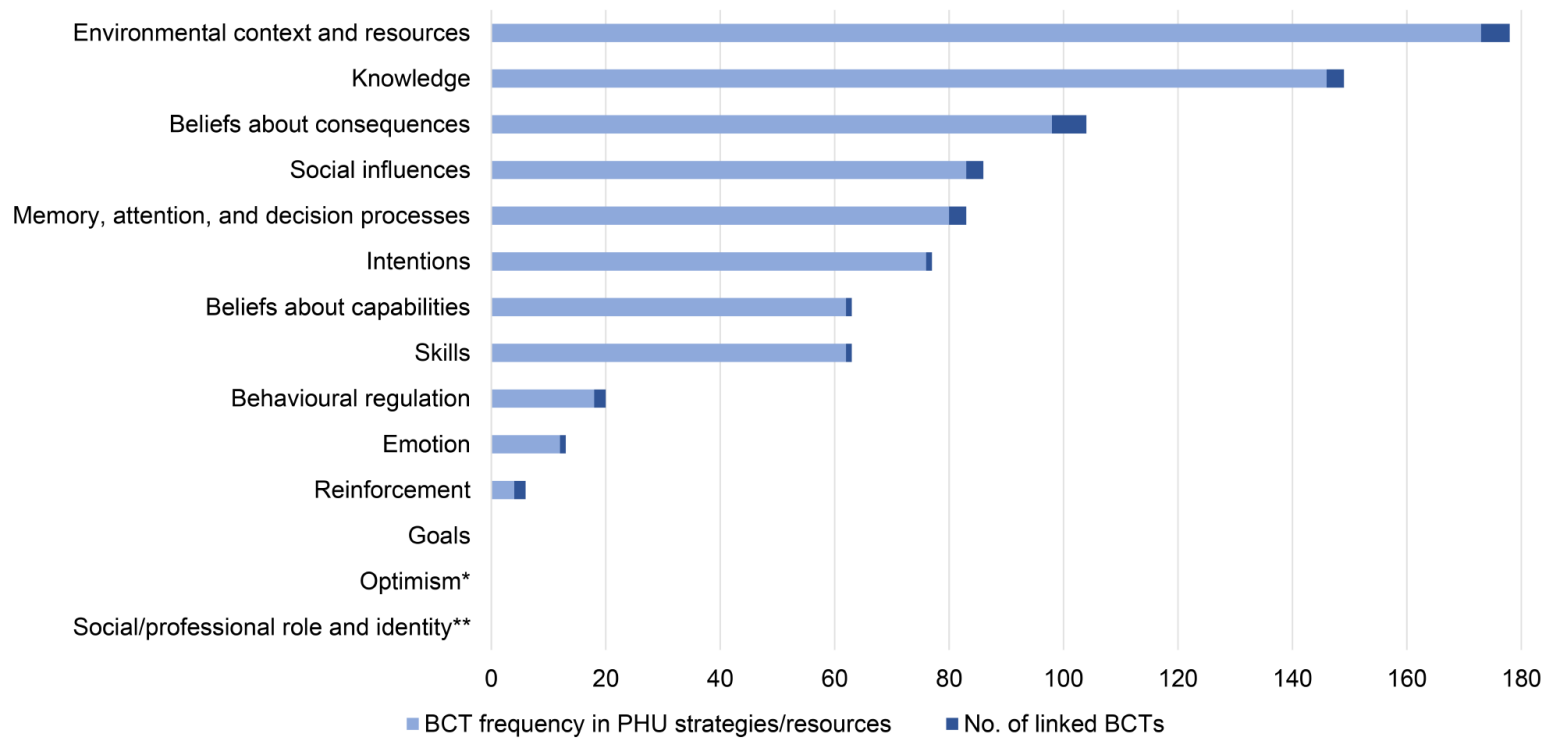
Frequency of theoretical domains of barriers/facilitators addressed by conclusively linked Behaviour Change Techniques (BCTs) used across public health unit (PHU) strategies/resources. *The Optimism domain was not linked to any identified BCTs. **Domain contained BCTs with inconclusive links to BCTs only. *Note*. BCTs with no evidence of links to the TDF are not included. See Table 2

Together, the PHUs addressed 11 of the 14 TDF domains through conclusively linked BCTs operationalised within their strategies/resources (Fig. 1).

Based on the BCTs used and how they there were operationalised, the TDF-based domains that were most frequently addressed overall were Environmental context and resources, Knowledge, and Social influences, followed by Beliefs about consequences. These domains were also addressed by more types of BCTs (i.e., 3 to 5 different linked BCTs) than the domains which were less frequently addressed (I.e., 2 or fewer different linked BCTs). For example, Environmental context and resources had 5 linked BCTs and Beliefs about consequences and Social influences had 4, while the Emotion and Skills domains each have a single linked BCT. Intention, Beliefs about capabilities, and Skills were also somewhat frequently addressed, with 1–2 different BCTs each.

The Goals and Social/professional role and identity domains were not conclusively linked to any BCTs found to be used by PHUs; however, they were inconclusively linked to 3 identified BCTs (*material reward*, and *social support (unspecified)* and *social comparison*, respectively) [17]. The Optimism domain was not inconclusively linked to any identified BCTs [17]. BCTs linked inconclusively only to domains were *social support (emotional)*,* generalisation of behaviour* and *credible source*.

## Discussion

This study investigated the online (websites and social media) and in-person strategies/resources promoting first or third doses of the COVID-19 vaccine used by three Ontario PHUs between January 2021 and May 2022 (online) and Winter 2023 (in-person). We mapped our findings to the TDF [9, 17] and BCTT [16, 18] to systematically investigate which BCTs are already being used within and across jurisdictions and where there may be opportunities for optimisation of further strategies and resources. We also investigated whether strategies/resources were tailored to the low-uptake groups that each PHU prioritised (Ottawa = adults in five low socioeconomic status neighbourhoods, Peel = Eastern European people between the ages of 30 and 49, Toronto = Black adults in five neighbourhoods with the lowest vaccination rates). PHUs varied in the degree to which they tailored strategies/resources to the prioritised groups (culturally and/or geographically); however, across PHUs we found notably fewer population-specific strategies/resources (and operationalised BCTs) than behaviour-specific (13–31 versus 39–79 strategies/resources, respectively), suggesting opportunities to further tailor existing strategies/resources/BCTs for priority groups.

Across PHUs, we identified 21 out of 93 BCTs used to address a range of barriers and facilitators to COVID-19 vaccination. This leaves many potentially applicable BCTs that have not yet been used, for example, the BCT *graded tasks* (linked to the Skills and Beliefs about capabilities domains), which has been effective at supporting health behaviours such as physical activity in adults with chronic health conditions [29–31]. In the context of vaccination, *graded tasks* could be operationalised through a step-by-step breakdown of the steps needed to receive a dose of the COVID-19 vaccine with boxes to tick as steps are completed, such as speak with a healthcare professional or community ambassador about (with a space to write questions), book an appointment, plan my journey to the appointment, and so on. Another example of an unused BCT is *pros and cons* (linked to the Beliefs about consequences domain), which has been used to support acceptance and uptake of COVID-19 [32, 33] and human papillomavirus vaccines [34, 35], particularly within the context of decision aids. *Pros and cons* could be operationalised using an infographic or other resource comparing the advantages and disadvantages of receiving a dose of the COVID-19 vaccine, highlighting the small risk of vaccine side effects and large benefit of COVID-19 vaccination. While all may not be applicable, there are likely opportunities to enhance vaccine uptake efforts by using additional BCTs linked to addressing specific barriers informed by evidenced tools from the behavioural sciences, such as the BCTT and TDF. The BCTs not already operationalised may inform parts of new strategies that PHUs could implement and tailor to prioritised groups and protective behaviours. Additionally, less frequently operationalised BCTs such as *salience of consequences* (e.g., Community members describing how seeing the impact COVID-19 infection had on themselves/loved ones made them decide to get vaccinated) may have a larger impact if they are applied more frequently [34–36].

Relatedly, TDF domains which were not as frequently addressed, such as Social professional role and identity, Emotion, Reinforcement, and Goals processes may represent unaddressed barriers or untapped facilitators to receiving the first or third dose of the COVID-19 vaccine that could be relevant to the prioritised groups. Though more research is needed to determine what types of interventions may be effective in supporting vaccination among minority groups, it is likely that successful interventions will be multifaceted and tailored to different communities [37]. The development of new and/or modified BCTs should be informed by additional research investigating the factors that affect the decision to get vaccinated against COVID-19 for members of prioritised/minority/equity-deserving groups (including available resources), ideally using a framework such as the TDF to elicit a comprehensive range of barriers and facilitators.

It is perhaps unsurprising that the Optimism domain was not identified in this review, as it has only been linked (inconclusively) to one BCT (*review outcome goals*), which was not found. Findings regarding the role of optimism in vaccine uptake have been mixed, with evidence that optimism can act as both a barrier or facilitator, possibly depending on what the individual is optimistic about (e.g., vaccines, pandemic ending soon) [38–40]. The effects of optimism on behaviour change within the context of a pandemic and vaccine uptake should be further explored.

Despite inconclusive or no evidence of links to the TDF, the identified BCTs *social support (emotional)*, *generalisation of behaviour*, and *credible source* should still be regarded as potentially effective strategies to address barriers and facilitators to vaccine uptake. Notably, the former two were identified at a very low frequency, while the latter (*credible source*) was very frequently found across all PHUs, especially in Toronto. Such widespread application of this inconclusively linked BCT in real-world settings suggests that current evidence may not fully capture the utility of certain strategies for changing behaviour, particularly for increasing vaccine uptake in priority groups. Indeed, research has found that vaccine support (e.g., information, encouragement) from a credible source such as a health care professional or another trusted individual like a faith leader or family member can be a strong facilitator of vaccine acceptance and uptake [41, 42]. *Credible source* appears to be a well-suited BCT to address mistrust, which is a factor found to be highly influential in equity-deserving groups such as those included in this review [25, 43, 44].

The potential utility of behavioural science for understanding and affecting acceptance and uptake of vaccines such as influenza [45–48], human papillomavirus [49–52], pneumococcal and shingles [53] has been well-documented, and its application to COVID-19 vaccines is increasingly being recognised by researchers [54–56] and health officials. In October 2020, the WHO published a report on behavioural considerations in relation to COVID-19 vaccination acceptance and uptake [57] that highlighted an enabling environment, social influences, and motivation as three key behavioural drivers of vaccine uptake (in addition to knowledge). In alignment with this, the three PHUs included in the present review have implemented numerous strategies/resources and BCTs that address barriers and enhance facilitators predominantly within the TDF domains of Environmental context and resources, Social influences, and Knowledge (as well as Beliefs about consequences). Our findings are also in alignment with Crawshaw and colleagues’ evidence synthesis [21] that identified Knowledge, Social influences, Beliefs about consequences and Environmental context and resources as predominant TDF domains within the 34 included studies with equity-deserving groups. Specifically, the strategies/resources used by PHUs frequently addressed barriers/facilitators related to concerns about vaccine safety/efficacy and risk of COVID-19 infection (Beliefs about consequences), mistrust (Social influences), and access (Environmental context and resources) [21].

Different barriers and facilitators to COVID-19 vaccine acceptance and uptake may be present for different groups or populations. For individuals who may already be motivated to receive a dose, barriers may include factors related to the Environmental context and resources and Social and professional role and identity domains, such as getting time off work, accessing public transit to a clinic, or finding childcare, or the Social influences domain, such as mistrust in government or public health authorities. Other barriers that might exist for motivated individuals could include not knowing how or where to get vaccinated or not speaking the language. For individuals who are less motivated to receive a dose of a COVID-19 vaccine, the barriers may include concerns about vaccine safety, side effects, and speed of development (Beliefs about consequences), fear or worries about side effects (Emotion), or previous negative experiences with other vaccines (Reinforcement). Many of these barriers are related to issues of access and trust, and may disproportionately affect priority groups and equity-deserving groups such as ethnic/racial minorities, migrants, and those included in this review [21, 58–60], and indeed, these are some of the barriers that the strategies used by the PHUs addressed (Table 2). Though there has been an increase in research investigating COVID-19 vaccine acceptance and uptake in equity-deserving groups, more evidence is needed to provide insight from these communities to improve public health programs.

In addition to COVID-19 vaccination, PHUs are also prioritising routine vaccination programs (e.g., measles, mumps, and rubella), which have seen a marked decrease since the COVID-19 pandemic (e.g., measles coverage dropped to 81% globally in 2021, the lowest level since 2008), causing health authorities to raise concerns [61, 62]. This may be primarily attributed to decreased and disrupted access during the pandemic. However, to date, coverage has largely still not returned its pre-pandemic rate, and other factors such as mistrust may be contributing [63, 64], particularly for equity-deserving groups who have experienced historical injustices by medical and government systems, lower participation in clinical trials, high costs of care, and importantly, lower access to healthcare services including vaccines [43]. Behavioural science can provide insight into the reasons why more people may be deciding not to get or are unable to get vaccinated post-pandemic compared to pre-pandemic and help to develop evidence-informed strategies/resources that PHUs can use to increase vaccine acceptance and uptake for routine vaccination programs.

The methodology of this study is likely transferrable to jurisdictions within or outside of Canada, within further support for future doses of COVID-19 vaccine and likely for routine immunization programs. It can be used by researchers to support understanding of the current and past state of public health efforts in support of goals other than vaccine uptake and for priority groups other than those identified by the PHUs in this study. Assessments of existing programs (online and in-person) using a common method to categorise both the strategies/resources and the barriers/facilitators can complement primary studies in behavioural science and clarify the content of past and present public health initiatives. This can serve to contextualize and enhance behavioural science-informed recommendations for future public health strategies and interventions, in a manner that complements rather than overlaps what PHUs are already doing or have already done.

While the WHO declared that the COVID-19 pandemic is no longer a global public health emergency in May 2023, COVID-19 continues to circulate widely in Canada. With this declaration, the WHO recommended that countries nevertheless ensure preparedness by maintaining efforts to increase COVID-19 vaccination coverage for people in high-priority groups and addressing vaccine acceptance and demand issues by working with communities to achieve inclusive risk communications, engagement, and interventions adapted to local contexts [6, 65]. Moreover, in November 2023, the WHO’s Strategic Advisory Group of Experts on Immunization (SAGE) released updated recommendations for the use of booster doses prioritizing high-risk groups [66]. Behavioural science is well positioned to support these endeavors and should be capitalised upon by public health programs in Canada and across the globe.

### Limitations

Data were collected primarily through online sources, then supplemented and checked by co-authors at each PHU. Nevertheless, our data capturing unpublished/in-person strategies/resources may be less comprehensive than our data on online strategies/resources. Although, given the focused assessment within specific groups within the cities, and with the involvement of PHUs, this risk is likely mitigated. Additionally, we did not have complete information about the ethnic demographics of the neighbourhoods that were prioritised by Ottawa. Therefore, data were collected pertaining only to strategies/resources that were tailored to the population geographically, not demographically. Lastly, for this review, we did not engage with members of the priority groups and thus local perspectives regarding what tailoring looks like for their communities may not have been fully captured. However, this review precipitated a larger project by our research team centered on enhancing understanding of COVID-19 vaccine uptake among the three priority groups through community partnerships and engagement [67].

### Future directions

By providing insight into the past and current strategies/resources of three urban Ontario PHUs through leveraging behavioural science tools, and highlighting addressed TDF domains and operationalised BCTs, this review has provided a launch point for future investigation. The next steps for the OPTimise Platform will be to continue working with PHUs and the communities they serve to design evidence-based recommendations for strategies to increase uptake of the COVID-19 vaccine within the prioritised groups.

## Conclusion

This study revealed additional opportunities for each of the PHUs to apply evidence from behavioural science to enhance and build upon their collection of strategies and resources to support uptake of the COVID-19 vaccines. PHUs should consider developing new and/or expanding current strategies/resources which are informed by the non- or less frequently operationalised BCTs and less frequently addressed TDF domains, especially those which have been identified as barriers to vaccination decisions and uptake. PHUs should also consider increasing the tailoring of strategies/resources to prioritised behaviours and groups, and partnering with and drawing from behavioural science may provide further opportunities for doing so.

## Electronic supplementary material

Below is the link to the electronic supplementary material.

Supplementary Material 1

Supplementary Material 2

## Data Availability

The datasets used and/or analysed during the current study are available from the corresponding author on reasonable request.

## Abbreviations

BCT: Behaviour Change Technique
BCTT: Behaviour Change Technique Taxonomy (version 1)
PHU: Public health unit
TDF: Theoretical Domains Framework
