# Supplementary material for: Strategies and resources used by public health units to encourage COVID-19 vaccination among priority groups: a behavioural science-informed review of three urban centres in Canada
Source: BMC Public Health. 2025 Jan 31;25:403. doi: 10.1186/s12889-025-21342-1 (PMC11786512; doi:10.1186/s12889-025-21342-1)
Supplement: Supplementary file 1 — Supplementary Material 1 [file 12889_2025_21342_MOESM1_ESM.docx]

Appendix 2

**Search Terms**

**Ottawa PHU:** ‘COVID-19’, ‘Vaccination’, ‘Vaccine’, ‘Third dose’, ‘3rd dose’, ‘Booster’, ‘Newcomer’, ‘Immigrant’, ‘Ledbury’ ‘Herongate’, ‘Parkwood Hills’ ‘Stewart Farm’, ‘Bayshore’ ‘Belltown’, ‘Hawthorne Meadows’ ‘Sheffield Glen’, ‘Emerald Woods’, ‘Sawmill Creek’.

**Peel Region PHU:** ‘COVID-19’, ‘Vaccination’, ‘Vaccine’, ‘First dose’ and ‘1st dose’, ‘Adult’, ‘Young Adult’, ‘30-49’, ‘Middle-Aged’, ‘Newcomer’, ‘Immigrant’, ‘Europe’, ‘Ukraine’, ‘Ukrainian’, ‘Poland’, ‘Polish’, ‘Russia’, ‘Russian’, ‘Eastern Europe’, ‘Eastern European’.

**Toronto PHU:** ‘COVID-19’, ‘Vaccination’, ‘Vaccine’, ‘First dose’, and ‘1st Dose’, ‘Black’, ‘African’, ‘Caribbean’, ‘Newcomer’, ‘Immigrant’, ‘Taylor Massey’, ‘Kingsview Village’, ‘Elms-Old Rexdale’, ‘Etobicoke West Mall’, ‘Englemount-Lawrence'.

*Note.* PHU = public health unit
