# Supplementary material for: Strategies and resources used by public health units to encourage COVID-19 vaccination among priority groups: a behavioural science-informed review of three urban centres in Canada
Source: BMC Public Health. 2025 Jan 31;25:403. doi: 10.1186/s12889-025-21342-1 (PMC11786512; doi:10.1186/s12889-025-21342-1)
Supplement: Supplementary file 2 — Supplementary Material 2 [file 12889_2025_21342_MOESM2_ESM.docx]

Appendix 2

**Data Abstraction Variables and Definitions**

Table 1. Data abstraction variables and definitions for public health unit (PHU) strategies/resources from online sources.

| **Column Heading** | **Definition** |
| --- | --- |
| Source | - Name of website/publishing body |
| Source URL | - Link to finding |
| Date accessed | - Date researcher extracted material from finding |
| Timestamp | - Date posted or updated |
| Title/Topic | - Title (or if no title, topic) of finding |
| External or Internal | - Is the finding from within or outside of the PHU of interest? (e.g., Peel PHU website = internal, link on PHU website to Ontario Public Health = external) |
| Link(s) to external | - If applicable, link to supporting URL of external entity |
| Deviation from PHU | - Number of deviations or “steps” finding is from the PHU website (e.g., if finding is on PHU website/social media page = 0, if finding is on the Ontario Public Health website via link on PHU website = 1, if finding is on Health Canada website via Ontario Public Health website via link embedded on PHU website = 2) |
| Format or mode of delivery | - How the finding is displayed for communication (e.g., text, video, webpage) |
| Description | - Brief detailing of the finding and its message(s) |
| Engagement evidence | - If applicable, include number of views, comments, shares, retweets, reactions, likes, etc. |
| Target-specific behaviour (y/n) | - Does the finding refer to the target behaviour? |
| Implicit or Explicit (I/E) | - Does the finding implicitly or explicitly refer to the target behaviour? |
| Evidence of target-specific behaviour | - Provide all relevant supporting text/material |
| Evidence location  (behaviour) | - Description of location in finding for supporting text/material (e.g., time in video, section of webpage) |
| Target-specific population (y/n) | - Does the finding refer to the target population? |
| Implicit or Explicit (I/E) | - Does the finding implicitly or explicitly refer to the target population? |
| Evidence for target-specific population | - Provide all relevant supporting text/material |
| Evidence location (population) | - Description of location in finding for supporting text/material (e.g., time in video, section of webpage) |
| Language(s) | - Indicate which language(s) the finding uses |
| Translated languages | - If applicable, indicate which languages are available and/or indication of translation/multilingual services |
| Lay (y/n) | - Is finding in basic language which does not require any post-secondary education to understand? |
| Directive (y/n) | - Does the finding contain a directive? (e.g., “Get your vaccine”) |
| Evidence location (directive) | - Description of location within finding for supporting text/material of directive (e.g., time in video, section of webpage) |
| Rationale (y/n) | - Does the finding provide support or rationale for the behaviour? (e.g., “Vaccines protect you and your community”) |
| Rationale evidence | - Provide all relevant supporting text/material |
| Evidence location (rationale) | - Description of location within finding for supporting text/material (e.g., time in video, section of webpage) |

*Note.* PHU = public health unit; (y/n) = yes/no.
